# Spondylodiscitis Caused by *Aspergillus* Species

**DOI:** 10.3390/diagnostics11101899

**Published:** 2021-10-14

**Authors:** Christos Koutserimpas, Ifigeneia Chamakioti, Symeon Naoum, Konstantinos Raptis, Kalliopi Alpantaki, Diamantis P. Kofteridis, George Samonis

**Affiliations:** 1 Department of Orthopaedics and Traumatology, “251” Hellenic Air Force General Hospital of Athens, 115 25 Athens, Greece; chrisku91@hotmail.com (C.K.); chamakiotiifigeneia@gmail.com (I.C.); naoumsimeon@gmail.com (S.N.); kraptis1981@hotmail.gr (K.R.); 2 Department of Orthopaedics and Traumatology, “Venizeleion” General Hospital of Heraklion, 714 09 Heraklion, Greece; apopaki@yahoo.gr; 3 Department of Internal Medicine, University Hospital of Heraklion, 715 00 Heraklion, Greece; kofteridisd@hotmail.com

**Keywords:** fungal vertebral infection, spondylodiscitis, *Aspergillus*, fungus, aspergillosis, osseous fungal infection

## Abstract

Background: Spondylodiscitis caused by *Aspergillus* spp. is a rare but life-threatening clinical entity. However, a consensus on diagnostic criteria and most effective medical management is still missing. The present study is a review of all published cases of spondylodiscitis caused by *Aspergillus* spp., in an effort to elucidate epidemiology, patients’ characteristics, andand the medical and surgical treatment options and their effectiveness. Methods: A thorough review of all existing spondylodiscitis cases caused by *Aspergillus* was performed. Data regarding demographics, responsible fungus, time between symptoms’ onset and firm diagnosis, antifungal treatment (AFT), surgical intervention, andand the infection’s outcome were investigated. Results: A total of 118 *Aspergillus* spondylodiscitis cases, yielding 119 *Aspergillus* spp. isolates, were identified in the literature. The patients’ mean age was 40.6 years. Magnetic resonance imaging (MRI) (after its introduction) indicated the diagnosis in most cases (66.7%), while definite diagnosis was established through cultures in the majority of cases (73.7%). *Aspergillus fumigatus* was isolated in most cases (73; 61.3%), followed by *Aspergillus flavus* (15; 12.6%) andand *Aspergillus nidulans* and *terreus* (7; 5.9%, each). The mean time between symptoms’ onset and diagnosis was 5.7 months. Amphotericin B was the preferred antifungal regiment (84 cases; 71.2%), followed by voriconazole (31; 26.3%), and the mean AFT duration was 6.1 months. The final outcome was successful in 93 cases (78.8%). Furthermore, 77 patients (65.3%) underwent surgery. Conclusions: Spondylodiscitis caused by *Aspergillus* spp. represents a clinical challenge, requiring a multidisciplinary approach. The present review has shown that prolonged AFT has been the standard of care of the studied cases, while surgical treatment seems to play an important role in selected patents.

## 1. Introduction

*Aspergillus* osteomyelitis represents a rare extra-pulmonary manifestation of invasive Aspergillosis, while vertebral Aspergillosis depicts the most common type of osseous infection by this mold [1]. Although rare, it represents a severe condition, often under- or misdiagnosed. Three different pathogenetic mechanisms have been proposed: direct invasion by contiguous pulmonary foci, hematogenous diffusion, and iatrogenic or traumatic inoculation [1,2].

The presenting symptoms of the infection are nonspecific, with backache being the predominant complaint. Vertebral Aspergillosis may be presented with osteolytic radiological findings, intervertebral disc lesions, and epidural space collections. Immunocompromised patients, such as solid organ recipients, patients with hematologic malignancies, those with severe neutropenia, and those receiving high-dose steroids are the more susceptible to invasive aspergillosis [1,3]. The disease may result in neurological deficit and spinal deformities [3].

Early diagnosis, achieved by cultures and/or histopathology after direct sampling and proper therapy are of paramount importance regarding diagnosis and management [1,3]. All patients require causative antifungal treatment (AFT) and many of them additional surgical intervention [1,2,3]. Few *Aspergillus* spondylodiscitis cases can be found in the literature. Therefore, a consensus on diagnostic criteria and the most effective medical management are based on limited data.

The present study is a review of all published cases of *Aspergillus* spondylodiscitis in an effort to describe epidemiology, patients’ characteristics, and medical and surgical treatment options and their effectiveness. The present review covers cases from vast geographical regions, and a long-time period. During the time covered by the present study, diagnostic methods and tools, as well as medical therapeutic managements, have changed dramatically.

## 2. Materials and Methods

A thorough electronic search in PubMed and MEDLINE databases was conducted to locate all existing articles related to cases of spondylodiscitis caused by *Aspergillus* spp. through July 2021. Alone and/or in combination the terms “fungal vertebral infection”, “fungal spondylodiscitis”, “fungal spine infection”, “vertebral aspergillosis”, “*Aspergillus* spine infection”, and “*Aspergillus* spondylodiscitis” were searched.

The review was limited to papers published in English and in peer-reviewed journals. Expert opinions, book chapters, studies on animals and on cadavers or in-vitro investigations, and abstracts in scientific meetings were excluded. Each selected case was confirmed to meet the criteria of *Aspergillus* spondylodiscitis. Each case had to have signs and/or symptoms of vertebral and/or disc infection, confirmed to be due to *Aspergillus*.

The data extracted and evaluated from these studies included age, gender, affected vertebral level, responsible *Aspergillus* spp., other sites with aspergillosis, co-infection with bacterial species, C-reactive protein (CRP) and erythrocyte sedimentation rate (ESR) at initial presentation, the presence of an immunosuppressive condition, time between symptoms’ onset and definite diagnosis (microscopy/culture/histopathology), duration and type of AFT, and type of surgical intervention. Furthermore, the results of medical and surgical treatment and follow-up of each case were studied.

Regarding AFT, it is of note that voriconazole, which was introduced in 2003, has been proven as the drug of choice against *Aspergillus* spp. Since the present review covers nine decades, a separate analysis of AFT after 2003 has been performed. Treatment was considered successful if all signs and symptoms of the infection had disappeared and no recurrence was observed during the follow-up period.

Data were recorded and analyzed using Microsoft Excel 2019 (Microsoft Corporation, Redmond, WA, USA).

## 3. Results

A total of 118 cases (86; 72.9% males), covering an 84-year period (1936–2020), were identified [2,4,5,6,7,8,9,10,11,12,13,14,15,16,17,18,19,20,21,22,23,24,25,26,27,28,29,30,31,32,33,34,35,36,37,38,39,40,41,42,43,44,45,46,47,48,49,50,51,52,53,54,55,56,57,58,59,60,61,62,63,64,65,66,67,68,69,70,71,72,73,74,75,76,77,78,79,80,81,82,83,84,85,86,87,88,89,90,91]. The studied population’s mean age was 40.6 years [standard deviation (SD) = 20.8].

The cervical spine was affected in four cases (3.4%), thoracic in fifty-two (44.1%) and lumbar in forty-four (37.3%), while the infection affected the cervical and thoracic spine in four cases (3.3%), thoracolumbar in seven (5.9%) and lumbosacral in five (4.2%). In one case (0.8%), the cervical, thoracic, and lumbar sections of the spine were affected, while in another one, the affected section was not available.

Additionally, 90 patients (76.3%) were immunocompromised, according to the available information from each report. Mean C-reactive protein (CRP) and erythrocyte sedimentation rate (ESR) at initial presentation were 98.5 mg/L (SD = 65.8) and 83.6 mm/h (SD = 25.6) respectively. The mean time interval between symptoms onset and firm diagnosis was 5.7 months (SD = 8.5). Another site of *Aspergillus* infection was present in 52 cases (44.1%). The mean follow-up was 16 months (SD = 12.1).

Imaging techniques performed for indicating diagnosis are presented in Table 1. Plain X-rays were performed on 80 patients (67.8%), followed by magnetic resonance imaging (MRI) in 42 (35.6%), computed tomography (CT) scans in 34 (28.8%), myelograms in 19 (16.1%), and bone scans in 11 (9.3%). In five cases (case 29, 95–98 in Table 1), no imaging was performed. As seen in Table 1, case 56 in 1994 represents the first one in which MRI was used, which is the gold standard of imaging for spondylodiscitis. Thus, after 1994, MRI was used in most cases (42; 66.7%), followed by plain X-rays (32; 50.8%), CT scans (18; 28.6%), bone scans (4; 6.3%) and myelograms (3; 4.8%).

Table 2 highlights the methods of definite diagnosis. Most cases of aspergillosis were diagnosed through cultures (87; 73.7%), followed by histopathology (50; 42.4%). Cultures and histopathology were performed in 20 cases (16.9%). PCR is reported in two cases (cases 89 and 113 in Table 2), while serology and/or galactomannan antigen is reported in four (cases 26, 79, 88, and 103 in Table 2).

A total of 119 *Aspergillus* spp isolates were yielded from 118 patients (case 30 in Table 2 yielded both *Aspergillus fumigatus* and *niger*). Seventy-three (61.3%) *Aspergillus fumigatus* were found, followed by fifteen (12.6%) *A. flavus*, seven (5.9%) *A. nidulans* and *A*. *terreus* each, two (1.7%) *A. versicolor*, and one (0.8%) *A. niger* and *A. calidoustus each*, while thirteen (10.9%) isolates were not further characterized. Furthermore, in eleven cases (9.3%) co-infection with bacteria was reported.

Regarding AFT, in sixty-one cases (51.7%), a single agent was used; in forty-three (36.4%) two agents were used, either simultaneously or consecutively; and in nine (7.6%) more than two agents were used. In five cases (4.1%) data regarding the specific antifungal drug were not reported (cases 2, 8, 11, 46, 47 in Table 2). Mean duration of AFT was 6.1 months (SD = 6.1).

Amphotericin B was the preferred agent in eighty-four cases [(71.2%), in thirty-eight (45.2%) as monotherapy], followed by voriconazole in thirty-one [(26.3%), in thirteen3 (42%) as monotherapy], itraconazole in twenty-eight [(23.7%), in eleven (39.3%) as monotherapy], flucytosine in nineteen [(16.1%), none as monotherapy], caspofungin in nine [(7.6%), none as monotherapy], miconazole in two [(1.7%), none as monotherapy], and fluconazole in one [(0.8%), not as monotherapy].

Regarding AFT in cases reported after 2003 [the introduction of voriconazole; a total of fifty cases (69–118 in Table 2)], in twenty-one cases (42%), a single agent was used; in twenty-two (44%), two, either simultaneously or consecutively, were used; and in seven (14%), more than two agents were used. The mean duration of AFT was 7.3 months (SD = 6.4). Voriconazole was the preferred agent in thirty-one cases [(62%), in nineteen (61.3%) as monotherapy], followed by amphotericin B in twenty-seven [(54%), in 3 (11.1%) as monotherapy], itraconazole in eighteen [(36%), in five (27.8%) as monotherapy], caspofungin in nine [(18%), none as monotherapy], flucytosine in two [(4%), none as monotherapy], and fluconazole in one [(2%), not as monotherapy].

During the 1936–2002 period, the outcome was successful in 55 cases (80.9%), while mortality rate was 17.6%. The outcome during the 2003–2020 period was successful in 38 cases (76%), while mortality rate was 20%. Finally, during the overall study period, the outcome was successful in 93 cases (78.8%), while a total of 22 patients passed away due to the infection (mortality rate = 18.6%).

A total of 77 patients (65.3%) underwent surgery for infection management. In five cases, the specific procedure was not mentioned (cases 3, 55, 96, 97, 98 in Table 2), while twenty-six patients underwent decompression and fusion (33.8%), twenty-five underwent debridement (32.5%), twenty underwent only decompression (26%), and one patient (1.3%) underwent only fusion. The outcome in patients receiving surgical and AFT was successful in 61 cases (79.2%), while the outcome in patients receiving only AFT was successful in 32 cases (78%).

## 4. Discussion

Fungal spondylodiscitis represents a rare and severe life-threatening clinical entity, requiring long-term medical, and, often, surgical treatment [1,3,83]. Aspergillosis represents a rare disease, as its incidence is 12 cases per year/1,000,000 people [91]. Therefore, data and information regarding spondylodiscitis caused by *Aspergillus* spp are scarce. The present study reviewed all published cases of spondylodiscitis caused by *Aspergillus* spp., in an effort to elucidate epidemiology, patients’ characteristics, and the medical and surgical treatment options and their effectiveness.

The present study reviewed 118 patients and 119 isolated *Aspergillus* species, with 16 months of follow-up, during an 84-year period. *Aspergillus* species are found worldwide in soil and decaying matter [76,80,92,93,94]. Invasive *Aspergillus* infections are most commonly observed in patients with significant underlying immunosuppression [92,93]. In the present review, most patients were immunocompromised (76.3%). The mean time interval between symptoms’ onset and diagnosis was 5.7 months. Fungal infections usually have an insidious onset with non-specific symptoms, and, therefore, diagnosis may be delayed [3,94,95].

Twenty different *Aspergillus* species may cause infections in humans. The most frequently isolated is *Aspergillus fumigatus*, while *A. flavus*, *A. niger*, and *A. nidulans* follow in frequency [93]. In the present review, *A. fumigatus* was predominant (61.3%), followed by *A. flavus* (12.6%) and *A. nidulans* and *A. terreus* (5.9% each). In all the reviewed cases, the causative *Aspergillus* had been isolated and recognized through cultures and/or histopathology and in two cases (case 79 and 89 in Table 2) through serology.

Diagnosis was based mainly in cultures, microscopy, and/or histopathology, while in most cases after 1994, diagnosis was indicated by MRI, and during the last few years, diagnosis was also based in a few cases on molecular techniques and serology (cases 26, 79, 88, 89, 103 and 113 in Table 2). It is of note that the present review covers almost nine decades, and it is thus understandable that identification techniques have evolved through time, ranging from simple microscopy, pathology, and cultures to modern molecular ones.

*A. fumigatus* is the most common and pathogenic species of *Aspergilli*. Resistance of *A. fumigatus* to antifungal agents, especially to azole compounds, represents a concern in the management of the disease [93,96,97,98]. Even though an increased rate of azole resistance has been reported recently in the Netherlands and the United Kingdom, due to the extensive use of azoles as pesticides, the prevalence of azole resistance reportedly remains low in other countries [93]. Thus, it is of paramount importance to perform identification and susceptibility testing to obtain accurate MIC values following the *Aspergillus* isolation. Ιt must be noted that according to EUCAST Antifungal Clinical Breakpoints from 2020 for a number of *Aspergillus* species, laboratory methods indicating MICs are standardized, while the immune status of the patient plays a major role [99,100].

Regarding AFT, the clinical importance of *Aspergillus* infection has increased as the number of immunocompromised patients has risen during the last decades [1,78,87,93]. Antifungals recommended for treatment of patients with invasive aspergillosis are triazoles and amphotericin B, with cidal action and echinocandins with static effect. Patients with such infections often require prolonged AFT [96].

Voriconazole, which was introduced in 2003, has been proven as the drug of choice against *Aspergillus* spp. [101] This agent has dramatically changed *Aspergillus* infections’ management during the last decades. This agent, having all the characteristics of azole compounds, is moderately hepatotoxic and much less nephrotoxic than all amphotericin compounds [66,78,96,102]. This is depicted in the results of the present study since, when taking into account the whole study period, amphotericin B was the preferred agent in 84 cases (71.2%), either as monotherapy or in combination with another antifungal agent, followed by voriconazole in 31 cases (26.3%), either as monotherapy or in combination with another antifungal agent. When taking into account only the published cases after 2003, voriconazole was the preferred agent in 31 cases (62%), followed by amphotericin B in 27 (54%). Amphotericin B represents an effective broad spectrum antifungal agent. However, it is relatively toxic, and its side effects, including renal dysfunction, may restrict its long-term use, which is essential for such infections [102]. The liposomal compounds of amphotericin B have reduced considerably the drug’s nephrotoxicity, but long use of these agents may still challenge the kidneys’ function [102]. Although the information about the type of amphotericin B was not available in most cases, it is assumed that during the last two decades, lipid or liposomal compounds of amphotericin B have been the drugs of choice due to the reduced side effects, as compared to deoxycholate amphotericin B.

The severity of *Aspergillus* infections may require also surgical intervention, as has occurred in some of the reviewed cases [3,61,83]. Vertebral instability, neurological deterioration, and the infection’s progression during the course of antifungal therapy should be closely observed and carefully considered as criteria for surgical intervention. The severity of these infections is also depicted in the mortality rate of the reviewed cases (18.6%).

The present review has some limitations. It covers a very long time period (almost nine decades), during which medical treatment has changed dramatically. Furthermore, not all data were available from each report, such as anti-fungal dosages, mode of administration, serum-levels monitoring, MICs, and complications. Nevertheless, this study reviews all the spondylodiscitis cases caused by *Aspergillus* spp., providing valuable information regarding epidemiology, severity, treatment, and outcome.

The present review has shown that spondylodiscitis caused by *Aspergillus* spp. represents a very challenging clinical entity, requiring a multidisciplinary approach since, in some cases, surgical intervention may be necessary. Proper medical AFT, based on susceptibility testing, when feasible, and surgical intervention, when required, seem to be the current standard management, while a prolonged period of AFT seems to be necessary. Fungal species should be routinely investigated in culture negative spondylodiscitis, especially in immunocompromised patients. Since these infections have poor prognosis and a relative high mortality rate, early diagnosis for targeted medical therapy is of utmost importance. More research and information are needed, since these infections are rare, focusing mainly on proper treatment.

## Figures and Tables

**Table 1:** Imaging techniques that each case underwent during the process of diagnosing the infection. The year 1994 represents the first report of MRI being used for diagnosis. CT: computer tomography, MRI: magnetic resonance imaging.

Period	X-ray	Myelogram	Bone Scan	CT	MRI
Before 1994	48 (87.3%)	16 (29.1%)	7 (12.7%)	16 (29.1%)	0 (0%)
After 1994	32 (50.8%)	3 (4.8%)	4 (6.3%)	18 (28.6%)	42 (66.7%)
Total study period	80 (67.8%)	19 (16.1%)	11 (9.3%)	34 (28.8%)	42 (35.6%)

**Table 2:** Definite diagnosis of spondylodiscitis caused by *Aspergillus* spp. NR: not reported. (Although the specimen (bone, tissue, abscess, etc.) studied is not reported, the technique is.)

Case No.	Method Used for Definite Diagnosis			
	Culture	Microscopy and/or Histopathology	Molecular Diagnostic Techniques/Other Tests	Minimal Inhibitory Concentration (MICs)
1.	abscess	-	-	-
2.	tissue specimen	-	-	-
3.	-	tissue specimen	-	-
4.	tissue specimen	-	-	-
5.	bone specimen	-	-	-
6.	thoracic mass	-	-	-
7.	tissue specimen	pulmonary infiltrate	-	-
8.	abscess	-	-	-
9.	disk space tissue	-	-	-
10.	abscess	-	-	-amphotericin B = 6 mg/L; -flucytosine > 250 mg/L.
11.	tissue specimen	-	-	-
12.	tissue specimen	-	-	-
13.	-	tissue specimen	-	-
14.	-	tissue specimen	-	-
15.	disk space tissue	-	-	-
16.	tissue specimen	-	-	-
17.	-	bone specimen	-	-
18.	tissue specimen	-	-	-
19.	abscess	-	-	-
20.	tissue specimen	-	-	-amphotericin B = 0.31 mg/L.
21.	tissue specimen	-	-	Testing for MIC indicated sensitivity to amphotericin B and 5-fluorocytosine.
22.	tissue specimen	-	-	Testing for MIC indicated sensitivity to amphotericin B and 5-fluorocytosine.
23.	bone specimen	-	-	-amphotencin B = 1.56 mg/L
24.	tissue specimen	-	-	-
25.	-	tissue specimen	-	-
26.	-	tissue specimen	serology	-
27.	abscess	tissue specimen	-	-
28.	tissue specimen	tissue specimen	-	-
29.	material from empyema	-	-	-
30.	abscess	-	-	-
31.	bone and tissue specimen	-	-	-
32.	abscess	-	-	-
33.	-	tissue specimen	-	-
34.	tissue specimen	-	-	-
35.	bone specimen	-	-	-
36.	-	tissue specimen	-	-
37.	-	tissue specimen	-	-
38.	tissue specimen	tissue specimen	-	-
39.	-	tissue specimen	-	-
40.	abscess	-	-	-
41.	disc and bone specimen	-	-	-
42.	tissue specimen	-	-	-
43.	-	tissue specimen	-	-
44.	-	tissue specimen	-	-
45.	tissue specimen	-	-	-
46.	NR	-	-	-
47.	NR	-	-	-
48.	tissue specimen	-	-	-
49.	-	tissue specimen	-	-
50.	-	tissue specimen	-	-
51.	tissue specimen	tissue specimen	-	-
52.	tissue specimen	-	-	-
53.	tissue specimen	-	-	-
54.	tissue specimen	-	-	-
55.	-	tissue specimen	-	-
56.	disc and fibrous tissues	-	-	-
57.	-	NR	-	-
58.	-	NR	-	-
59.	tissue specimen	-	-	-
60.	tissue specimen	-	-	-
61.	-	tissue specimen	-	-
62.	tissue specimen	-	-	-
63.	abscess	-	-	-
64.	tissue specimen	-	-	-
65.	tissue specimen	tissue specimen	-	-
66.	tissue specimen	-	-	-
67.	-	tissue specimen	-	-
68.	tissue specimen	-	-	-
69.	tissue specimen	-	-	-
70.	NR	NR	-	-
71.	NR	NR	-	-
72.	NR	NR	-	-
73.	tissue specimen	-	-	-
74.	-	bone specimen	-	-amphotericin B = 0.25 mg/L; -fluconazole, > 256 mg/L; -5-fluorocytosine = 64 mg/L; -itraconazole = 0.5 mg/L; -voriconazole = 0.5 mg/L.
75.	tissue specimen	-	-	-
76.	bone and tissue specimen	-	-	-
77.	tissue specimen	-	-	-
78.	-	tissue specimen	-	-
79.	-	-	serological tests and high concentrations of *Aspergillus galactomannan* were detected in two consecutive serum samples with a commercial assay	-
80.	NR	NR	-	-
81.	NR	NR	-	-
82.	NR	NR	-	-
83.	NR	NR	-	-
84.	-	tissue specimen	-	-
85.	abscess	-	-	-
86.	abscess	-	-	-
87.	abscess	-	-	-
88.	-	disc specimen	serology	-
89.	-	-	(1) comparative analysis of the nucleotide sequence using the BLAST sequence analysis tool (2) polymerase chain reaction (PCR) from a bone tissue sample.	-
90.	abscess	-	-	-
91.	tissue specimen	tissue specimen	-	-
92.	abscess	-	-	-
93.	tissue specimen	tissue specimen	-	-
94.	-	tissue specimen	-	-
95.	NR	NR	-	-
96.	NR	NR	-	-
97.	NR	NR	-	-
98.	NR	NR	-	-
99.	NR	-	-	-
100.	abscess	-	-	-
101.	tissue specimen	-	-	-
102.	tissue specimen	-	-	-
103.	tissue specimen	-	galactomannan antigen test	-
104.	tissue specimen	tissue specimen	-	-
105.	-	tissue specimen	-	-
106.	-	bone specimen	-	-
107.	bone specimen	-	-	-
108.	bone specimen	-	-	-
109.	-	tissue specimen	-	-
110.	-	tissue specimen	-	-
111.	-	bone specimen	-	-
112.	bone specimen	-	-	-amphotericin B = 2 mg/L; -voriconazole = 0.75 mg/L; -caspofungin = 0.032 mg/L.
113.	disc specimen	-	polymerase chain reaction (PCR)	-amphotericin B = 2 mg/L; -caspofungin = 4 mg/L; -itraconazole = 1 mg/L; -posaconazole = 2 mg/L; -voriconazole = 4 mg/L; -terbinafine = 0.5 mg/L.
114.	abscess	-	-	-
115.	bone specimen	-	-	-
116.	-	tissue specimen	-	-
117.	tissue specimen	-	-	-
118.	abscess	-	-	-

## Data Availability

Not applicable.
